# Resistive Switching and Synaptic Characteristics in ZnO/TaON-Based RRAM for Neuromorphic System

**DOI:** 10.3390/nano12132185

**Published:** 2022-06-25

**Authors:** Inho Oh, Juyeong Pyo, Sungjun Kim

**Affiliations:** Division of Electronics and Electrical Engineering, Dongguk University, Seoul 04620, Korea; inho194@gmail.com (I.O.); joozero0908@gmail.com (J.P.)

**Keywords:** memristor, resistive switching, low and high current, bilayer, ZnO, TaON

## Abstract

We fabricated an ITO/ZnO/TaON/TaN device as nonvolatile memory (NVM) with resistive switching for complementary metal-oxide-semiconductor (CMOS) compatibility. It is appropriate for the age of big data, which demands high speed and capacity. We produced a TaON layer by depositing a ZnO layer on a TaN layer using an oxygen-reactive radio frequency (RF) sputtering system. The bi-layer formation of ZnO and TaON interferes with the filament rupture after the forming process and then raises the current level slightly. The current levels were divided into high- and low-compliance modes. The retention, endurance, and pulse conductance were verified with a neuromorphic device. This device was stable and less consumed when it was in low mode rather than high mode.

## 1. Introduction

In the era of artificial intelligence (AI) technology, nonvolatile memory (NVM) with high speed and density should be developed [1,2]. The most common NVM in the market, silicon (Si)-based flash memory, is used primarily because of its high density [3]. Scaling down the size of NVM to have a higher density has evolved through the photolithography process but reached its physical limits [4]. The end of Moore’s law is already just around the corner. Consequently, it is necessary to develop new memory devices to replace conventional memory [5]. Over the past few decades, new NVM types, such as resistive random-access memory (RRAM), phase change memory (PCM), magnetic random access memory (MRAM), and ferroelectric memory (FeRAM), have emerged [6,7,8,9]. RRAM is one of the most prominent candidates for new memory because of its high scalability [10], low power consumption [11], and high compatibility with complementary metal-oxide-semiconductor (CMOS) technology [12].

RRAM devices can be used as synaptic devices in neuromorphic computing, which has emerged as a solution to overcome the von-Neumann bottleneck problem [13]. Neuromorphic computing, which mimics the working mechanism of the human brain, is receiving significant attention [14]. Unlike von-Neumann architecture, neuromorphic computing requires low energy consumption because it consists of many connections that connect neurons and synapses in parallel [15]. RRAM is a so-called memristor, which can imitate the role of a synapse. For the RRAM to be used as an artificial synapse, multilevel operation, a high off/on ratio, and high reliability are required [16]. The conductance of RRAM, which corresponds to the synaptic weight, can be manipulated by applying voltage pulses [17,18,19]. Multiple conductance states can be gradually increased or decreased; these are similar to potentiation and depression in a biological system, respectively [20]. Linear and symmetric conductance changes are required to improve pattern recognition accuracy [21].

An RRAM device consists of a simple metal-insulator-metal (MIM) structure. An insulating oxide layer is sandwiched between two metal electrodes [22]. Because of this simple structure, RRAM can be used for a high-density cross-point array or three-dimensional (3D) integration [23]. There are binary states in memory storage: “0” and “1”. “0” denotes a state in which data are not stored, and “1” denotes a state in which data are stored. Data storage states depend on the resistance state: low-resistance state (LRS) or ON state and high-resistance state (HRS) or OFF state. “0” corresponds to HRS, and “1” corresponds to LRS. The device resistance can be switched by applying external voltage stress on the electrode.

The RRAM cell is initially in the HRS, which consists of applying high voltage stress because a soft breakdown is needed to switch HRS to LRS [24]—called the “forming process”. The “reset” process makes it possible to switch the RRAM cell from LRS to HRS by applying the “reset” voltage [22,25], and “set” makes it possible to switch the RRAM cell from HRS to LRS by applying the “set” voltage. The switching mechanism is based on the growth of conductive filament (CF). The CF is a path that connects the top electrode (TE) and bottom electrode (BE) of the RRAM cell. In the set process, CF is connected and becomes LRS, whereas in the reset process, CF is disconnected and becomes HRS.

Compliance current (CC) should be applied when performing a set transition. The CC limits the current as desired, prevents the permanent breakdown of the device, and adjusts the size of the CF. The process of reading data by applying a read voltage that does not affect the current state of the cell is performed to verify whether the cell is currently in the LRS state or the HRS state [26].

Various materials have been used in RRAM structures. Resistance switching characteristics vary depending on which material is used for the metal electrode and insulating layer. The use of transparent indium tin oxide (ITO) for the metal electrode is the most promising because of its high electrical conductivity [27]. TaN is also used in metal electrodes. It has tremendous reactivity when reacting with oxygen and oxidizes to TaON [28]. The formation of this interfacial layer can help resistive switching characteristics [29,30].

Various materials are used in the insulating layer, and metal oxides are one of the most promising types. Binary metal oxides, such as Al_2_O_3_ [31,32], NiO [33], TiO_2_ [34], HfO_2_ [35], and ZnO [36,37], have been studied because of their high compatibility with CMOS technology, high reliability, and simple material composition. ZnO is an n-type semiconductor with a wide bandgap (~3.37 eV at 300 K) [38], high transparency, many oxygen vacancies [39], and sensitivity to ultraviolet (UV) light. For example, ITO/ZnO/TiN devices have demonstrated that the phenomenon of resistance switching varies depending on the illumination of UV [40,41,42]. Transparent RRAM for invisible devices based on ZnO have recently used transparent electrodes such as ITO [43]. ITO/ZnO/ITO devices have high transmittance, reliability, and potential as synaptic devices [44].

## 2. Materials and Methods

The all-fabrication process of ITO/ZnO/TaN was conducted on a SiO_2_/Si substrate. The ~100 nm thick TaN, namely BE, was deposited by direct-current (DC) sputtering under argon (Ar) and nitrogen gas at room temperature, in which the working pressure was 5 mTorr. Then, the ~20 nm ZnO thin film as a switching layer was also deposited by DC sputtering. The zinc target was sputtered with Ar (6 sccm) and O_2_ (14 sccm) under 1 mTorr at room temperature. Finally, the TE ITO film with a thickness of ~100 nm was deposited via a shadow mask for 100 μm diameter by an e-beam evaporator.

X-ray photoelectron spectroscopy (XPS, KIST, Seoul, Republic of Korea) was used to analyze the compound elements of the device. Moreover, transmission electron microscopy (TEM, KANC, Suwon, Republic of Korea) with focused ion beam (FIB) milling was performed to identify the cross-section of the device cell. The electrical characteristics of the ITO/ZnO/TaN device were evaluated using a semiconductor parameter analyzer (Keithly 4200-SCS and PMU ultrafast mode, Tektronix Inc., Beaverton, OR, USA) in the voltage linear sweep mode; the pulse mode is customizable. The increasing step of voltage for set or reset switching was 0.05 V. Unlike DC mode, the electrical signals of pulse were consisted of zero state or set points that lead to change the conductance of device. Finally, A voltage bias was applied to all ITO TE devices, whereas the TaN BE device was grounded.

## 3. Results and Discussion

**Figure 1:**
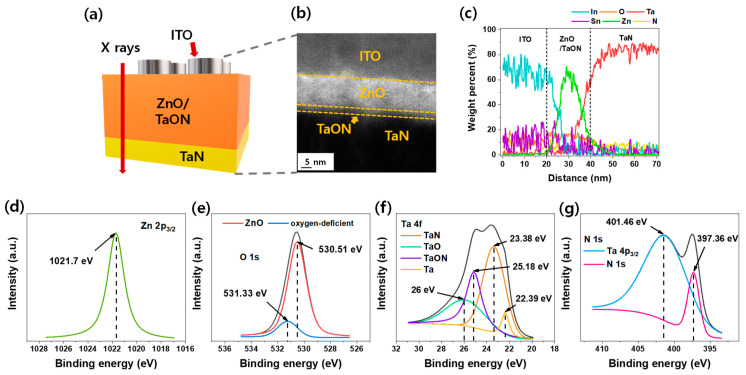
(**a**) Schematic of structure and components of ITO/ZnO/TaON/TaN device. (**b**) TEM image and (**c**) EDS weight percent. XPS spectra of (**d**) Zn 2p3/2, (**e**) O 1s, (**f**) Ta 4f, and (**g**) N 1s scan ZnO/TaON/TaN without TE.

Figure 1 confirms whether the desired stack is well-fabricated before investigating the characteristics of the ITO/ZnO/TaN device. Figure 1a is the device schematic. TEM and energy dispersive X-ray spectroscopy (EDS) analyses were performed from ITO to TaN, and XPS was performed to investigate the ZnO/TaON interface more closely. The TEM image in Figure 1b indicates that the dark area is the TaN layer, the white area is the ZnO layer, the gray area is the ITO layer, and a TaON layer between ITO and ZnO is observed. All elements of each layer were detected by the EDS weight percent, depicted in Figure 1c.

In the ITO TE layer, indium (sky blue) occupies the most weight, but tin (purple) and oxygen (orange) occupy the smallest portion. Oxygen has a light weight at 16 g/mol, which is much less than the 114.8 g/mol of indium. Tin is 118.7 g/mol but accounts for a much smaller percentage than indium in the ITO e-beam source. The weight percent of each element of the ITO is depicted in Figure 1c. In the ZnO/TaON switching layer, zinc (green), oxygen (orange), tantalum (red), and nitrogen (yellow) are observed. Nitrogen also has a light weight of 14 g/mol, so it appears small. The thickness of the ZnO/TaON layer is approximately 20 nm, confirmed by EDS, as expected.

The X-ray was used with Ar^+^ etching for the ZnO/TaON/TaN section for XPS depth mode to probe the ZnO and TaON chemical response. Figure 1d,e illustrates the peak position of Zn 2p_3/2_ and O 1s in bulk ZnO. Moreover, Figure 1f,g illustrates Ta 4f and N 1s in the ZnO/TaN interfacial layer, respectively. The reported peak binding energy of Zn 2p3/2 is near 1021 eV, which is similar to the observed 1021.7 eV. This peak energy indicates the oxidized Zn state, but another metallic state of Zn was not found [45].

The spectra of O 1s revealed two peaks at 530.51 and 531.33 eV. The low binding energy peak, 530.51 eV, is associated with the reaction of Zn and O. Another peak, 531.33 eV, is related to oxygen-deficient regions in bulk ZnO [46]. In Figure 1e, the binding energy peaks for metallic Ta and TaN correspond to 22.39 and 23.38 eV, respectively [47]. The positions at 25.18 and 26 eV are attributed to TaON and Ta_2_O_5_, respectively [48]. The spectra of N 1s have two peaks located at 401.46 and 397.36 eV. The high and low energies are ascribed to Ta overlapping this section of the N 1s spectrum and to metal TaN, respectively [49].

Furthermore, the zinc target was sputtered by only Ar and oxygen gas when the ZnO layer was deposited on the TaN layer. Nevertheless, the result of EDS and XPS illustrate that the TaON layer exists. The TaON layer could be created when the TaN layer is oxidized. Oxygen sources can be from the ZnO layer as deposited using the Ar and oxygen gas during sputtering. While the ZnO was deposited, a negative bias was applied to the target, and a positive bias was applied to the substrate by DC sputtering; thus, the Ar+ ions were moved toward the target, and the O^−^ ions were moved toward the substrate. In this process, the oxygen plasma collided with the surface of the TaN, penetrated into the TaN layer, and formed a new TaON layer.

Researchers in a previous study experimented with moving oxygen vacancies and oxygen ions in the surface and bulk of the ZnO layer using different oxygen plasma treatment times [50,51]. The conditions under which ZnO was deposited were O_2_ of 14 sccm and time longer than 60 s; thus, the oxygen plasma effect could be significant on the TaN surface. This device was intentionally fabricated as an ITO/ZnO/TaN device but was unintentionally produced as a bi-layer with the ITO/ZnO/TaON/TaN device.

**Figure 2:**
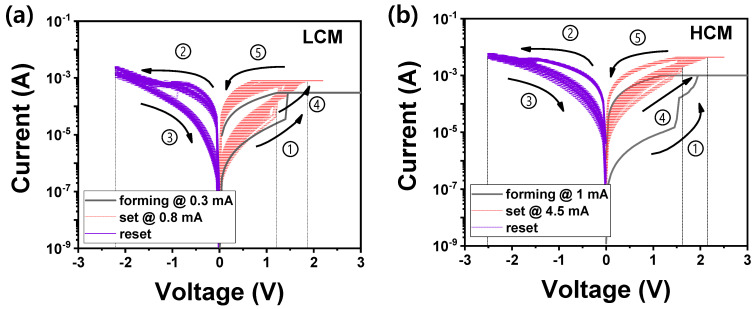
I−V curves of ITO/ZnO/TaON/TaN RRAM in (**a**) LCM and (**b**) HCM. Black lines indicate forming process, purple lines indicate reset process, and red lines indicate set process. The one cycle switching follows the order of the numbers (①→⑤).

The I−V characteristics of this device were measured by dividing it into two cases—(a) low CC mode (LCM) and (b) high CC mode (HCM)—as depicted in Figure 2. In the initial device state, electroforming (black line), in which electrons can flow freely, must be induced by applying a voltage bias to the TE and ground to the BE. Electroforming switching was performed at 0.3 mA in LCM and 1 mA in HCM, respectively, to prevent permanent breakdown that degrades the device’s electric characteristics. Then, the device that changes from HRS to LRS was applied with a reset voltage to the TE, returning the transition from LRS to HRS for ON/OFF switching.

In both the LCM and HCM, the resistance of HRS before electroforming is larger than the resistance of HRS after reset switching. The TaON layer, which was additionally created, forming a bi-layer with ZnO, prevents the device from returning to the initial HRS before forming switching [52]. The additional layer causes more interfacial defects where the charges can be easily trapped, increasing the overall conductance of the device. A larger reset voltage applied to the device to completely rupture the CF path increases the permanent breakdown probability. Therefore, the reset voltage does not increase, but rather the CC increases above the forming CC to improve the OFF/ON ratio.

In Figure 2a (LCM), the initial device applies ① 4 V at a CC of 0.3 mA to turn on the device. The device should apply a ②, ③ −2.2 V sweep without CC to return the device off state. This voltage is low because the higher reset voltage can break down the device, so it cannot fully rupture the CF path. In contrast, based on the 0.1 V reading of the LCM, the resistance of HRS before forming switching is 163 kΩ, and the resistance of HRS after reset switching is 67.1 kΩ. This result confirms that –2.2 V is not enough voltage to return to the initial device state. The device applies ④, ⑤ positive voltage at 0.8 mA CC to switch to the on state again. The CC of set switching is higher than forming switching, which improves the window (i.e., OFF/ON ratio). In the LCM case, the set voltage (not applying voltage but turning on the device) is distributed from 1.3 to 1.85 V.

As depicted in Figure 2b (HCM), it is a similar process as in performed in LCM, but the magnitude differs. The CC is increased from 0.3 mA; the CC of the LCM is ① 1 mA for forming the filament in the initial device state. ②, ③ The reset voltage with −2.5 V is higher than when the reset voltage is applied in LCM. As the CC increases, more oxygen vacancies are created. Therefore, a large voltage should be applied for recombining oxygen vacancies and ions but not larger than the breakdown voltage.

The LCM set switching is performed by increasing less from 0.3 (forming) to 0.8 (set) mA, whereas the HCM is performed by increasing by even more, from ④, ⑤ 1 to 4.5 mA—another result of increasing the CC. The reset switching is difficult because of the high CC. The CC difference between forming and set switching must be larger than the LCM to improve the window. The resistances of HRS at 0.1 V in forming and reset switching are 304 and 3.43 kΩ, respectively. Eventually, the resistance does not return to the initial state after reset switching. In this case, the set voltage is between 1.7 to 2.15 V.

When comparing the LCM and HCM, the ratios of CC increasing from forming to set process are 260% (0.3 mA → 0.8 mA) and 350% (1 mA → 4.5 mA), respectively. Although the HCM has a large ratio, the LCM has a large window size. Furthermore, for power (set and reset voltage), the HCM is 1.7 to 2.15 V and −2.5 V, while the LCM is 1.3 to 1.95 V and −2.2 V. Consequently, the power consumption of HCM is higher than LCM. Despite the large reset voltage in HCM, the reset efficiency is low. The LCM-HRS changed from 163 to 67.1 KΩ, and the HCM-HRS changed from 304 to 3.43 kΩ. The LCM operates more effectively than HCM in power consumption, window efficiency, and reset efficiency.

**Figure 3:**
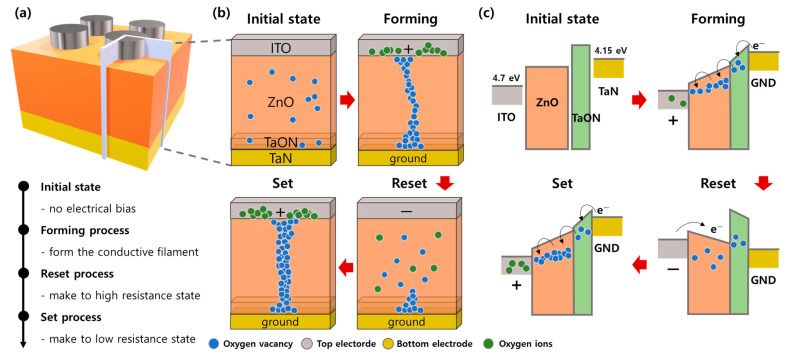
Schematic of switching process and mechanism. (**a**) ITO/ZnO/TaON/TaN schematic. (**b**) Movement of oxygen ions and oxygen vacancies by electrical force. (**c**) Energy band diagram of the device for each process.

Figure 3 is the schematic for the switching mechanism of the ITO/ZnO/TaON/TaN device. An initial device to which an electrical force is not applied cannot form the CF inside the ZnO-TaON switching layer. The oxygen vacancies/ions that switch the device into the LRS are activated and moved in ZnO/TaON layer by electrical forming bias. The transferred oxygen ions are absorbed by ITO when the positive bias applies to the TE. The ITO material accept oxygen ions efficiently, producing oxygen vacancies when the oxygen ions move.

The oxygen vacancies left by oxygen ions moving are depicted as blue spheres in Figure 3b. The oxygen vacancies function as electron acceptors, increasing conductance. A negative bias was applied to the TE to induce the oxygen ions to recombine with oxygen vacancies to return to HRS. Although the filament is ruptured, it is not completely ruptured by the TaON layer, suggesting it does not return to the same HRS as the initial state. Consequently, when set switching proceeds at CC, such as in the forming process, a small window is formed by the HRS, whose current level increases. Therefore, the set switching proceeds with a higher CC, and a thicker filament is formed than in the forming process, as depicted in Figure 3b Set.

The conduction mechanism is also explained by energy band diagrams. The work function of the ITO and tantalum nitride electrodes are approximately 4.7 and 4.15 eV, respectively [53,54]. The electron affinity of zinc oxide is higher than tantalum oxynitride [42]. Therefore, the energy band diagram of ITO/ZnO/TaON/TaN is represented in Figure 3c in its initial state. The positive bias for the conductive path that consists of oxygen vacancies applies to ITO, and the free electrons can flow from TaN to ITO through the oxygen vacancies [43]. This phenomenon is the Poole–Frenkel emission effect [55], which describes how an electric current flows efficiently despite the trapping of electrons in an insulator when a sizeable electric force is applied to the TE.

The thermal fluctuations provide energy to an electron to help remove it from the oxygen vacancies and into the conduction band. However, in a large electric field, the electron does not need energy because the field leads the electrons. The oxygen vacancies recombine with oxygen ions provided from ITO by applying the negative bias to ITO. The electrons have difficulty flowing forward to ground, indicating that the device returns to the HRS. The electrical force applies to the TE, like the forming process, to turn on the device. However, in this case, for the window, the CC should be higher because the TaON layer has many defects that are hard to remove with the reset process.

**Figure 4:**
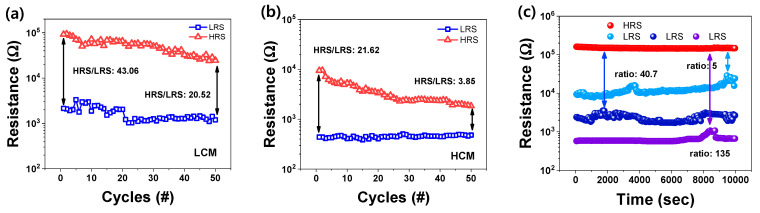
Endurance in (**a**) LCM and (**b**) HCM. (**c**) Retention of four levels controlled by CC at set switching.

Figure 4 illustrates the endurance, which measures 50 cycles of HRS and LRS. The read voltage was set to 0.2 V to distinguish between LRS and HRS more than 10 times. For the endurance cycle of LCM in Figure 4a, HRS varied from 92.02 to 24.45 kΩ, LRS varied from 2.14 to 1.20 kΩ, and the HRS/LRS ratio varied from 43.06 to 20.52. In both LRS and HRS, the resistance decreased as the cycle progressed, and the variation of HRS is larger than that of LRS, caused by the continuous depletion of oxygen ions deposited on the electrodes during reset switching. The lack of oxygen ions reduces the possibility of recombination of oxygen vacancies, which results in insufficient reset and reduces HRS resistance.

For the endurance cycle for HCM in Figure 4b, HRS varied from 9.45 to 1.85 kΩ, LRS varied from 0.48 to 0.44 kΩ, and the ratio of HRS/LRS varied from 21.62 to 3.85 kΩ. Comparing Figure 4a,b, the uniformity in HCM was higher in LRS, but the HRS and HRS/LRS ratios were higher in LCM. Therefore, the LCM may perform a more reliable operation in the switching process.

In Figure 4c, the four retention states were measured with different CCs—depending on the CC, four states are distinguishable. One HRS and three LRS exist without significant variation between 0 and 10,000 s. Based on HRS, the HRS/LRS ratios for low CC, middle CC, and high CC are 135, 40.7, and 5, respectively. The HRS/LRS ratio was calculated by dividing the minimum of HRS by the maximum of LRS. Multilevel conduction is required for a synaptic device to obtain a high-density memory capacity. Accordingly, the ITO/ZnO/TaN devices demonstrated that it is possible to maintain multilevel data storage over time.

Next, depression was performed to imitate the synapse characteristics, including potentiation. A linear increase and decrease in conductance are required to mimic a synaptic network. The ITO/ZnO/TaN device applied a 50-pulse train with a constant amplitude to LCM at 2.55 V/10 μs to potentiation and −2.1 V/10 μs to depression. The amplitude of the read pulse was 0.2 V. The potentiation part revealed that conductance increased abruptly, which functions similarly to the abrupt increase of current during the set switching in the initial I–V curve of DC mode in Figure 2a. Under these pulse conditions, conductance could be adjusted in a range between 198 and 732 μS.

Likewise, in HCM, a 50-pulse train with a constant amplitude applied 1.85 V/200 μs to potentiation and −2.1 V/200 μs to depression. As depicted in Figure 2b, during set switching in the I–V curve, the current gradually increases compared with Figure 2a. Thus, the conductance of the potentiation is more gradually increased than LCM. Conductance varied from 631 to 1251 μS under these pulse conditions. Unlike the linear potentiation of the change in conductance except for the first pulse in the LCM, the gradual potentiation of the change in conductance is observed in HCM. In the depression area, both LCM and HCM decreased gradually. Excluding the first pulse, it is easier for LCM to predict the conductance variations than HCM.

**Figure 5:**
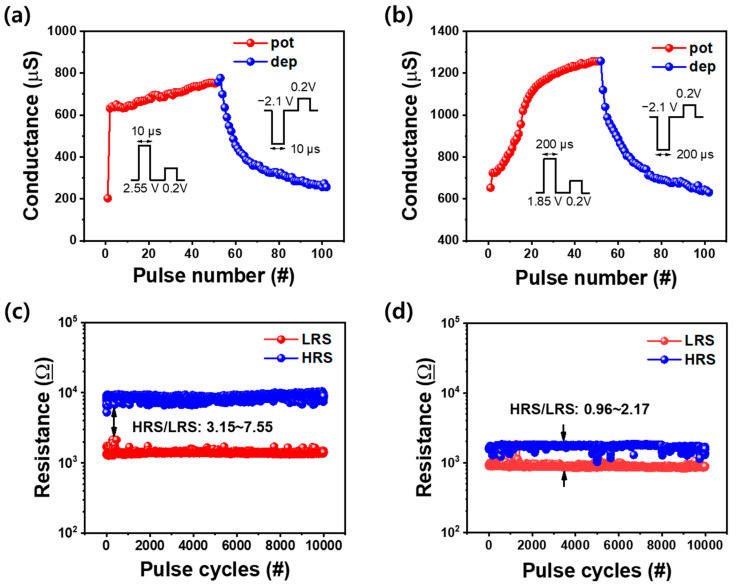
Conductance change in potentiation (red)/depression (blue) by (**a**) the small electric force and (**b**) the large electric force. The resistance change in the 10,000 pulse cycles by (**c**) the small electric force and (**d**) the large electric force.

We conducted pulse endurance, as depicted in Figure 5c,d. The pulse width and interval were set to 10 and 200 μs, respectively. A read voltage of 0.2 V was applied, and 10,000 set/reset pulse cycles were measured. In LCM, both LRS and HRS were observed as uniform. The pulse endurance was smaller than the DC endurance for HRS because the current decreased less during the reset process in pulse mode. However, uniformity was more stable than DC mode in both LRS and HRS. In HCM (unlike in LCM), because of the smaller HRS, the uniformity decreased as in the I–V curve for the HRS/LRS ratio but was more stable than in DC mode. In pulse mode, even though HCM and LCM both have high uniformity in pulse endurance, their size is larger to distinguish ON and OFF states in LCM. Thus, LCM has superior synaptic properties to HCM.

## 4. Conclusions

We investigated the conduction mechanism and synaptic characteristics of an ITO/ZnO/TaN device. First, the device stack was confirmed by SEM, TEM, EDS, and XPS analyses. Because of the TaON layer, which can cause more interfacial defects, a larger CC than the electroforming process was required in the set process to improve the OFF/ON ratio. LCM operated more reliably than HCM based on comparing the I–V curves, endurance tests, and retention. Moreover, by varying the set CC, multiple-level resistance states were achieved over time. We demonstrated potentiation and depression—required to mimic synaptic networks—by applying a constant amplitude pulse train. In pulse mode, it was easier to predict conduction variations in LCM, with a larger OFF/ON ratio. Accordingly, LCM is more suitable for neuromorphic devices.

## Data Availability

Not applicable.
